# Phase 1 safety, tolerability, pharmacokinetics and pharmacodynamic results of KCL‐286, a novel retinoic acid receptor‐β agonist for treatment of spinal cord injury, in male healthy participants

**DOI:** 10.1111/bcp.15854

**Published:** 2023-08-09

**Authors:** Maria B. Goncalves, Tim Mant, Jörg Täubel, Earl Clarke, Hana Hassanin, Daryl Bendel, Henry Fok, John Posner, Jane Holmes, Adrian P. Mander, Jonathan P. T. Corcoran

**Affiliations:** ^1^ Neuroscience Drug Discovery Unit The Wolfson Centre for Age‐Related Diseases, King's College London, Guy's Campus London UK; ^2^ NIHR Biomedical Research Centre at Guy's and St Thomas' NHS Foundation Trust and King's College London, Guy's and St Thomas' NHS Foundation Trust London UK; ^3^ Richmond Pharmacology Limited London UK; ^4^ Surrey Clinical Research Centre University of Surrey Surrey UK; ^5^ Centre for Pharmaceutical Medicine Research, Institute of Pharmaceutical Science King's College London London UK; ^6^ Nuffield Department of Primary Care Health Sciences University of Oxford Oxford UK; ^7^ Centre for Trials Research Cardiff University Cardiff UK

**Keywords:** KCL‐286, pharmacokinetics, RARβ agonist, safety, spinal cord injury, target engagement

## Abstract

**Aims:**

KCL‐286 is an orally available agonist that activates the retinoic acid receptor (RAR) β2, a transcription factor which stimulates axonal outgrowth. The investigational medicinal product is being developed for treatment of spinal cord injury (SCI). This adaptive dose escalation study evaluated the tolerability, safety and pharmacokinetics and pharmacodynamic activity of KCL‐286 in male healthy volunteers to establish dosing to be used in the SCI patient population.

**Methods:**

The design was a double blind, randomized, placebo‐controlled dose escalation study in 2 parts: a single ascending dose adaptive design with a food interaction arm, and a multiple ascending dose design. RARβ2 mRNA expression was evaluated in white blood cells.

**Results:**

At the highest single and multiple ascending doses (100 mg), no trends or clinically important differences were noted in the incidence or intensity of adverse events (AEs), serious AEs or other safety assessments with none leading to withdrawal from the study. The AEs were dry skin, rash, skin exfoliation, raised liver enzymes and eye disorders. There was an increase in mean maximum observed concentration and area under the plasma concentration–time curve up to 24 h showing a trend to subproportionality with dose. RARβ2 was upregulated by the investigational medicinal product in white blood cells.

**Conclusion:**

KCL‐286 was well tolerated by healthy human participants following doses that exceeded potentially clinically relevant plasma exposures based on preclinical in vivo models. Target engagement shows the drug candidate activates its receptor. These findings support further development of KCL‐286 as a novel oral treatment for SCI.

**What is already known about this subject**

- Retinoic acid receptor (RAR) β signalling is involved in central nervous system (CNS) regeneration.
- RARβ is a neuronal transcription factor that regulates numerous pathways in CNS regeneration; these include, axonal outgrowth, synaptogenesis, modulation of the glial scar and axonal pathfinding.
- KCL‐286 is a preclinical RARβ agonist developed to treat CNS injuries.

**What this study adds**

- KCL‐286 was tested in healthy male adults to determine the safety, tolerability, pharmacokinetics and pharmacodynamics of this agent under investigation for the treatment of spinal cord injury.
- Safety and pharmacokinetic data indicate that KCL‐286 is well tolerated at a 100‐mg daily dose, which equates to a dose shown to elicit axonal regeneration in proof‐of‐concept models of spinal cord injury.
- KCL‐286 engages its receptor in white blood cells and maintains activation during dosing.
- KCL‐286, as far as we are aware, is the first orally available RARβ agonist drug for treatment of nerve injuries.

## 1 INTRODUCTION

The reported global prevalence of spinal cord injury (SCI) is between 0.7 and 1.2 million incident cases per year with falls and road accidents being the major causes.^1^ There are no licensed drugs that can overcome the intrinsic failure of the adult central nervous system (CNS) to regenerate or mitigate the associated comorbidities of SCI. The cost in the USA alone is estimated at $4 billion per year and is therefore, a substantial unmet clinical need.^2^ SCIs are heterogenous in terms of location and extent of tissue damage^3^ but have core commonality in terms of cellular and molecular pathologies that have been well characterized both in rats and humans. There is axonal damage, if not complete severance of the axons, cell death, inflammation and neuropathic pain.^4^, ^5^

**FIGURE 1:**
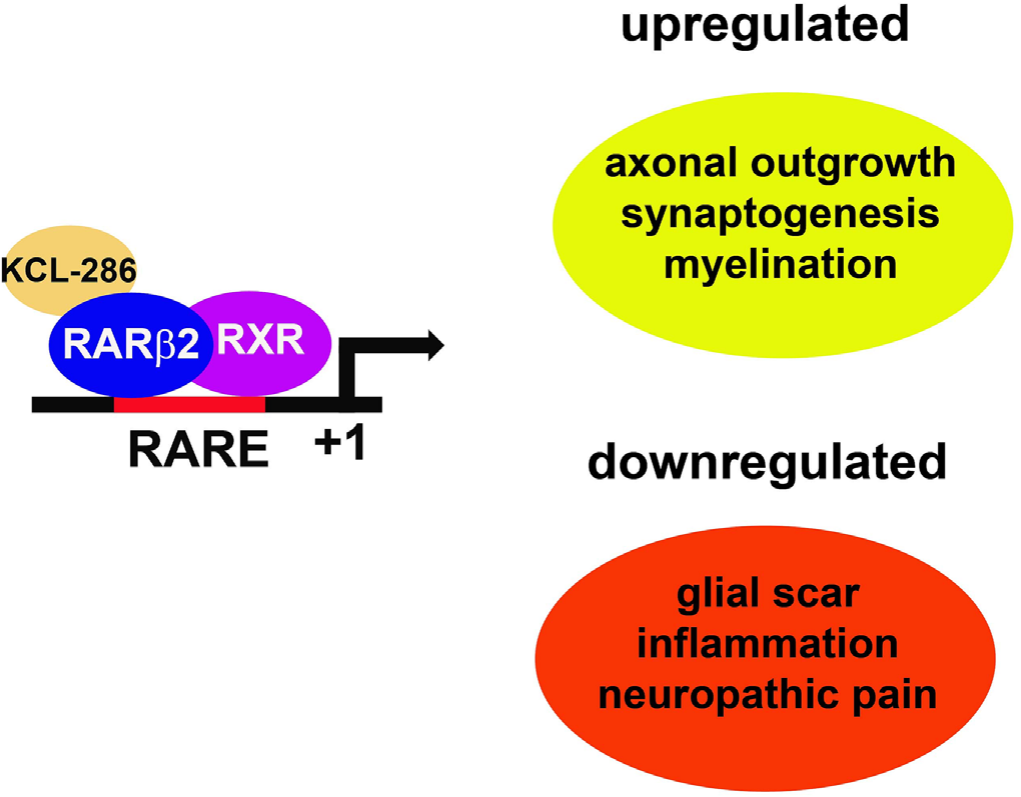
KCL‐286 transcriptional neuronal signalling. KCL‐286 binds to a retinoic acid receptor (RAR)β2/retinoid x receptor (RXR) heterodimer located at a retinoic acid response element (RARE). This results in activation of transcriptional pathways required for axonal regeneration.

The retinoic acid (RA) signalling pathway has been shown to be involved in axonal outgrowth. The pathway consists of the nuclear receptors, the RA receptors (RARs) and the retinoid x receptors (RXRs) of which there are 3 types—α, β and γ—and various subtypes. An RAR associates with an RXR and the heterodimer binds at RA response element (RARE) of target genes and transcription occurs once a retinoid binds (Figure 1).^6^ There are 4 subtypes of RARβ, 1–4^7^ and it is RARβ2 that is involved in neurite outgrowth/axonal regeneration in both lower and higher vertebrates.^8^, ^9^, ^10^, ^11^, ^12^, ^13^ RARβ2 is unique amongst the RARβ subtypes as it is autoregulated by its own agonist due to the location of a RARE in the promotor of the RARβ2 gene.^14^

KCL‐286, also known as C286, is an oral RARβ agonist with multifactorial reparative effects in the CNS.^15^, ^16^ Preclinical data have shown that mean values of t_1/2_ of the investigational medicinal product (IMP) were 1.4 h in rat and 2.5 h in dog.^15^ Brachial plexus avulsion is the most severe form of axotomy.^4^ Using a rat model of repair of where the sensory roots are severed and reimplanted into the SC, also causing SCI, it has been demonstrated that KCL‐286 induces axonal regeneration of both spinal and sensory nerves through the inhibitory environment of the CNS, modulates neuroinflammation and extracellular matrix molecules. It achieves this by activating RARβ2 in the injured neurons, which is induced in response to injury (Figure 1).^15^, ^16^ The anticipated benefit of KCL‐286 is to promote motor and sensory function following SCIs due to its multifactorial nature in altering pathways that are involved in nerve injury. The ideal treatment is to administer the drug 1–10 days after SCI, before the secondary cascade that results in tissue loss,^3^ allowing sustained axonal growth by feedback mechanisms^15^, ^17^ once the axonal growth is initiated during the 28 days of drug administration. Efficacy in humans would be a significant advance in treatment of SCIs. Due to its multimodal mechanism of action, KCL‐286 is predicted to have clinical applications in other CNS disorders such as stroke, traumatic brain injury, diabetic neuropathy, neuropathic pain and multiple sclerosis, where RA signalling has been shown to also have a role in regeneration.

To determine the risk: benefit ratio of KCL‐286 it is necessary to establish the safety and pharmacokinetic (PK) profile in healthy volunteers and then progress to establish efficacy in SCI patients. Here we report a Phase I safety and PK study of KCL‐286 and show that it is a safe and well‐ tolerated drug with a satisfactory PK profile that engages with its receptor in white blood cells (WBCs), where RARβ2 is expressed,^18^ resulting in a dose‐related upregulation of RARβ2.

## 2 MATERIAL AND METHODS

### 2.1 Study oversight

The present study is a phase 1, first‐in‐human double blind, randomized, placebo‐controlled trial, in male healthy volunteers, consisting of a single ascending dose (SAD) adaptive design with a food interaction (FI) arm, and a multiple ascending dose (MAD) design, to evaluate the safety, tolerability, PK and pharmacodynamics (PD) of KCL‐286. (Trial ID: https://www.isrctn.com/ISRCTN12424734, registration date, 18/07/2018).

The trial, protocol amendments, informed consent forms, investigator's brochure and any other relevant information were reviewed and approved by the London‐Surrey Borders Research Ethics Committee. All procedures performed in studies involving human participants were in accordance with the ethical standards of the institutional and/or national research committee and with the 1964 Declaration of Helsinki and its later amendments or comparable ethical standards.

The trial was conducted from July 2018 to December 2021 at 2 sites in the UK. All participants provided written informed consent.

### 2.2 Study design

This was a prospective, Phase I, double blind, randomized, placebo controlled, dose escalating study conducted at 2 sites. The study comprised 2 parts: Part A included SAD in 8 cohorts (S1 to S8) with a food interaction (FI) arm of 1 cohort and Part B MAD in 5 cohorts (M1 to M5) in which the volunteers were dosed once daily for 7 days after fasting overnight. For all cohorts, sentinel dosing was used, the first 2 participants, 1 active and 1 placebo in each cohort were first dosed and then the remainder of the cohort including 1 placebo were dosed up to 7 days later. Safety and interim plasma PK data were reviewed at safety review committee meetings prior to commencement of the next dose. There were at least 7 days between treatment periods.

The primary objective was to determine the safety and tolerability of KCL‐286 administered to healthy adult male subjects in SAD and MAD cohorts, the secondary objective was to determine PK The exploratory objective was to show target engagement of KCL‐286 in MAD cohorts by assessment of RARβ2 expression in WBCs. These data were not used to inform dosing decisions as it was an exploratory biomarker.

### 2.3 Study participants

Healthy adult males aged between 19 and 45 years were randomized into the trial. Female volunteers were excluded from this study as at that time reproductive toxicology data were not complete in female models. Participants had a body mass index ≥18 and ≤30 kg/m^2^ and body weight ≥60 kg and ≤90 kg at screening. Each participant signed a written informed consent form before undergoing comprehensive screening procedures at the clinical trial facility, including full medical history inquiry, full physical examination including neurological examination, vital signs, electrocardiogram and laboratory tests. The inclusion/exclusion criteria were the same for all parts of the study. Participants were instructed not to take any prescribed medications without informing the staff and agreed not to take any over the counter medications or herbal supplements (except for maximum of 4 g paracetamol per 24 h) for 2 weeks before screening and for the whole study duration, to maintain current dietary habits, had not used a sunbed within 3 months before screening and would not for the duration of the study and for 1 month after final study visit, agreed to remain abstinent from unprotected sexual intercourse for the duration of the study (from the time of the first dose until 3 months after the last dose). Participants were under continuous medical supervision during each admission period. Safety assessments were made at screening and regular intervals throughout each period until the end of follow‐up.

### 2.4 Sample collection and analysis

Blood and urine samples for PK analysis were collected from the individuals in all groups within 2 h before dosing and at 0.5, 1 1.25, 1.5, 2, 3, 5, 8, 12, 24, 36 and 48 h after dosing. Venous blood samples were collected in heparin treated tubes, and centrifuged within 30 min (400 × g, 4°C, 10 min). The upper plasma layer was collected, aliquoted and stored at −70 ± 10°C. Urine samples were aliquoted and stored at −70 ± 10°C. For RARβ2 expression in WBC from MAD cohorts, blood was collected before dosing and on Days 1, 2, 4, 7, 8, 9 and 14 and analysis carried out as described in supplementary material. This was carried out at the end of the MAD dosing. Analysis was conducted (York Biolabs) using a validated analytical method based on protein precipitation. High‐performance liquid chromatography–tandem mass spectrometry was used (API5000) to analyse the concentrations of KCL‐286 in blood and urine, over a concentration of 0.500–1000 ng/mL and a 50‐mL sample aliquot. The overall precision for the Quality Control samples at the Low QC, Med QC and High QC concentrations was 8.1, 3.8 and 2.7%, and the bias was 2.7, 2.2 and −0.8%, respectively. All below the level of quantification values were set to lower limit of quantification at 0.5.

### 2.5 PK assessments

#### 2.5.1 Determination of drug concentrations and dose proportionality

Plasma and urine PK parameters of KCL‐286 were analysed using noncompartmental analysis. Actual dosing and PK sampling times were used for PK parameters. Linear log trapezoidal method (linear on ascending and log on descending concentrations) was used to integrate the plasma concentration time profiles out to the time that the last measurable concentration. Parameters included, AUC, area under the plasma concentration–time curve; AUC (_0‐inf_), AUC from dosing time extrapolated to infinity; AUC (_0‐t_), AUC from dosing time to last observation; CL/F, clearance over F (bioavailability); C_max_, maximum observed concentration, occurring at T_max_; CV, coefficient of variation; λz, first order rate constant associated with the terminal (log‐linear) portion of the curve; T_lag_, time prior to the first measurable (nonzero) concentration; T_max_, time of C_max_; Vz/F, volume of distribution associated with the terminal phase divided by F (bioavailability); Ae, KCL‐286 excreted unchanged in a collection period; Ae_0‐t_, KCL‐286 excreted unchanged to the last measurable collection point; Ae%, percent of the dose excreted unchanged. No data imputation was performed. The mean concentration and associated descriptive statistics were calculated using quantifiable levels. Concentrations below the limit of quantitation before C_max_ were treated as zero. The PK parameters are summarized per cohort using descriptive statistics.

Dose proportionality was assessed using a power model which was fitted by restricted maximum likelihood using SAS Proc Mixed. The intercept and slope were fitted as fixed effects based on the assumption of linearity. An estimate of the slope was obtained from the power model along with its 2‐sided 90 and 95% CI. Exposure was deemed dose‐proportional if it was within 2‐fold of the mean.

### 2.6 Starting dose and escalation decisions

Safety results and limitations imposed by the protocol were the primary consideration when deciding on the starting dose and subsequent dose for the next cohort. To calculate the starting dose, the no‐observed‐adverse‐effect level from Day‐28 dog toxicokinetic study was used for planned ceiling exposure and dose level. The values were C_max_ 3700 ng/mL, AUC up to 24 h (AUC_0–24_) 25 110 ng h/mL at a dose of 140 mg. The minimum pharmacologically active dose (PAD) was 0.3 mg/kg in the rat, resulting in an average plasma exposure of C_max_ 138 ng/mL and AUC_0–24_ 911 ng h/mL. On a dosage basis, using the scaling factor of 6.2 for rats, the human equivalent dose for a 60‐kg study participant is 2.9 mg per participant (0.3 mg/kg/6.2*60 kg). Using the PAD methodology, there was approximately a 3‐fold margin between the proposed starting dose of 1 mg and the minimal PAD in rats on a dosage basis. On the more relevant exposure basis, there was a minimum of an 8‐fold safety margin. As there were no adverse events (AEs) from this dose, the next dose chosen was 2 mg as this was still within safety margins. In both the SAD and MAD parts of the study, after the second cohort had been treated and after every cohort thereafter, interim analyses were carried out using Bayesian linear regression models to help guide the dose‐escalation decisions. AUC_0–24h_ and C_max_ were independently modelled as functions of dose, assuming a linear relationship on the log scale for both the outcome and the dose, with noninformative priors. For the MAD part, PK data on Days 1 and 7 were modelled using a Bayesian hierarchical regression and included Day 1 data from the SAD study. At every dose‐escalation decision, the posterior distribution of the dose–exposure curve was used to predict the probability that a person in the next cohort would not exceed the predefined PK limits. If this probability was <.05 for both AUC_0–24h_ and C_max_ the next highest dose level was suggested for the next cohort of participants. An example of Bayesian modelling used to predict dose for MAD cohort 5 is described in supplementary material and Table S1.

### 2.7 Statistical methods

The data processing and statistical analysis of the results were performed by Richmond Pharmacology Limited. The PK parameters were computed using WinNonlin™ PK software (version 6.3 or higher Pharsight Corp, Sunnyvale, CA, USA). All other analyses were conducted using SAS PC Version 9.4 or later (SAS Institute, NC, USA). Quantitative data are expressed as the mean ± standard deviation.

### 2.8 Nomenclature of targets and ligands

Key protein targets and ligands in this article are hyperlinked to corresponding entries in http://www.guidetopharmacology.org, and are permanently archived in the Concise Guide to PHARMACOLOGY 2019/20.^19^

## 3 RESULTS

### 3.1 Subjects

A total of 109 healthy male participants took part in the study. The SAD substudy comprised 56 randomized participants: 42 participants across the single dose KCL‐286 treatment arms and 14 participants in the placebo arm. Across the cohorts, participants randomized to KCL‐286 included 3 to 1 mg, 4 to 2 mg, 5 to 48 mg and 6 participants each to 4, 8, 12, 24 and 100 mg. The 2‐mg cohort planned to include 3 participants treated with KCL‐286; however, 1 participant randomized to 2 mg KCL‐286 withdrew consent and was replaced. The 48‐mg cohort included 1 fewer participant randomized to KCL‐286 than planned (as agreed by the safety review committee). Therefore, 55 participants completed the SAD substudy.

Part A: FI (single dose crossover with 7‐day washout). For the FI substudy, a dose of 6 mg KCL‐286 was selected based on the data from the SAD. The dose was chosen to ensure exposure did not exceed previous safe doses assuming a 2‐fold increase in bioavailability following food. The FI cohort comprised 8 randomized participants as planned, 4 to the fasted–fed crossover arm and 4 to fed–fasted crossover arm. One participant from each FI arm discontinued the study: 1 in the 6‐mg fasted–fed arm discontinued due to an AE and use of prohibited medication and 1 in the 6‐mg fed–fasted arm discontinued due to noncompliance and was lost to follow‐up. The remaining 6 participants in the FI cohort (3 participants in each arm) completed the study as planned.

Part B: MAD (6 or 7 days of dosing once per day, fasted condition). This part of the study comprised 40 randomized participants: 30 across the multiple dose KCL‐286 treatment arms and 10 in the placebo arm. Across the cohorts, participants randomized to KCL‐286 included 6 each to the 6, 12, 24, 72 and 100‐mg multiple dose treatment arms. All 40 participants completed the MAD substudy as planned. The Surrey Clinical Research Centre had to close in December 2019; at this time, 6 SAD cohorts and the FI cohort were completed. Partially completed were SAD Cohort S7 (7 out of 8 participants dosed) and MAD Cohort M1 (5 out of 8 participants dosed). The trial was continued at Richmond Pharmacology, once covid restrictions allowed. The decision was taken to repeat MAD Cohort M1 in full (8 participants) to ensure that the full cohort was from the same study site. Furthermore, to reduce the potential for site bias in the analyses, 5 participants from the Surrey site randomized to Cohort M1 (4 randomized to active treatment and 1 randomized to placebo) were removed from the safety population.

The demographics of the participants are summarized in Table 1. These characteristics were similar across the SAD, FI and MAD substudies and across the treatment arms. One hundred and 4 participants were analysed for safety and tolerability and PK. Forty participants were analysed for RARβ2 expression from the MAD cohorts. The placebo dosing is not shown in the data analysis as these were below the level of detection.

**TABLE 1:** Summary of participant demographics.

Demographics	SAD (*n* = 56)	FI (*n* = 8)	MAD (*n* = 40)
Age (years), mean (SD)	28.6 (7.86)	24.6 (5.90)	26.3 (5.22)
Body mass index (kg/m^2^), mean (SD)	23.9 (2.387)	23.01 (2.940)	23.79 (2.801)
Height (cm), mean (SD)	177.6 (6.76)	179.8 (7.74)	177 (7.54)
Weight (kg), mean (SD)	75.46 (9.235)	74.74 (14.258)	74.42 (8.824)
Ethnicity			
Hispanic or Latino	3.6	12.5	7.5
Not Hispanic or Latino, *n* (%)	96.4	87.5	92.5
Race, *n* (%)			
Asian	5.4	0	5
Black or African American	7.1	12.5	2.5
Caucasian	82.1	62.5	90
Other	5.4	25	2.5

Abbreviations: FI, food interaction; MAD, multiple ascending dose; SAD, single ascending dose; SD, standard deviation.

### 3.2 SAD

**FIGURE 2:**
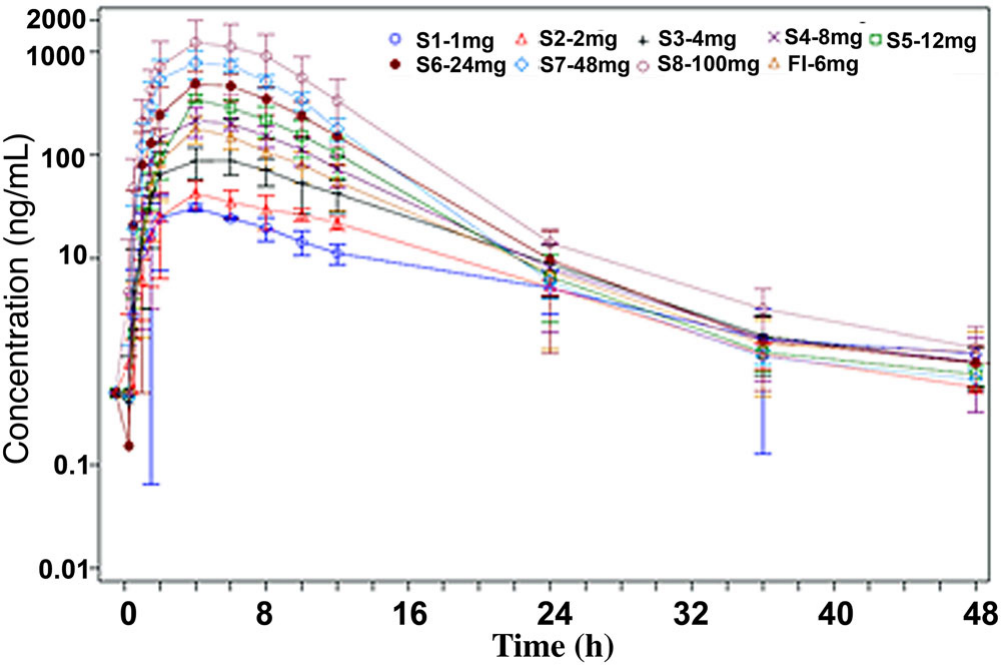
Overlaid mean plasma KCL‐286 concentration *vs.* nominal time (log‐linear scale) by treatment group (pharmacokinetic set)—single ascending dose (SAD) and food interaction (fasted). Error bars denote standard deviation.

Plasma concentration–time profiles overlaid for each dose group in the SAD (linear) are in Figure 2, and a summary of PK parameters for SAD cohorts in Table 2 and Table S2.

**TABLE 2:** Summary of pharmacokinetic parameters at Day 1 (PK set)—single ascending dose.

Parameter	Unit	Treatment dose				
Plasma		S4, 8 mg (*n* = 6)	S5, 12 mg (*n* = 6)	S6, 24 mg (*n* = 6)	S7, 48 mg (*n* = 5)	S8, 100 mg (*n* = 6)
AUC_0__‐inf_, D1	h ng/mL	2234.8(653.15)	2984.2 (712.2)	4706.9 (892.74)	6961.6 (1035.96)	11 416.1 (6842.98)
AUC_0‐t_ D1	h ng/mL	2226.7 (653.47)	2976.5 (712.61)	4701.4 (893.47)	6957.1 (1036.44)	11 407.4 (6839.13)
Cl/F	L/h	3.981 (1.7001)	4.245 (1.1584)	5.256 (1.0046)	7.023 (1.0864)	4.7138 (43.2)
C_max_ D1	ng/mL	244.2 (48.51)	351.2 (35.53)	527.5 (132.53)	832.6 (164.65)	1289.8 (737.26)
T_1/2_ D1	h	4.063 (1.1613)	3.706 (0.8683)	3.96 (0.8259)	4.48 (2.0162)	4.343 (0.9082)
λz D1	1/h	0.1828 (0.0524)	0.1963 (0.0483)	0.1819 (0.03984)	0.1736 (0.0541)	0.1643 (0.02713)
Tlag D1	h	0.38 (0.137)	0.33 (0.129)	0.21 (0.189)	0.21 (0.116)	0.17 (0.129)
T_max_ D1	h	4.003 (1.7926)	4.683 (1.0591)	1.0954 (21.9)	1.0237 (21.6)	4.339 (0.8138)
Vz/F obs	L	21.797 (5.5232)	21.881 (4.3757)	30.63 (10.7239)	21.9956 (48.5)	32.3109 (46.8)
AUC_0–24h_ D1	h ng/mL	2167.9 (609.86)	2929.2 (686.08)	4621.4 (892.28)	6905.3 (1035.14)	11 275.1 (6810.22)
CL/F (norm)	L/h kg	0.141 (0.0502)	0.055 (0.015)	0.075 (0.0213)	0.097 (0.0129)	0.141 (0.0502)
Vz/F (norm)	L/kg	0.298 (0.0682)	0.283 (0.0368)	0.435 (0.1819)	0.635 (0.3302)	0.897 (0.3952)
Urine						
Ae	ng	124 (225.68)	305.1 (263.37)	932.6 (928.67)	1266.5 (809.68)	2131.7 (1489.31)
Ae_(0–24h)_	ng	144.78 (292.48)	252.76 (222.868)	869.5 (958.863)	1287.3 (836.279)	2131.75 (1592.64)

Abbreviations: Ae, KCL‐286 excreted unchanged in a collection period; Ae_0‐t_, KCL‐286 excreted unchanged to the last measurable collection point; Ae%, percent of the dose excreted unchanged; AUC, area under the plasma concentration‐time curve; AUC (_0‐inf_), AUC from dosing time extrapolated to infinity; AUC (_0‐t_), AUC from dosing time to last observation; C_max_, maximum observed concentration, occurring at T_max_; CL/F, clearance over F (bioavailability); CV, coefficient of variation; D, days; h, hours; T_lag_, time prior to the first measurable (nonzero) concentration; T_max_, time of C_max_; Vz/F, volume of distribution associated with the terminal phase divided by F (bioavailability); λz, first order rate constant associated with the terminal (log‐linear) portion of the curve.

Data show mean and standard deviation in brackets.

In concentration–time data, there was a short lag time between dosing to appearance of KCL‐286 in plasma which became quantifiable at 0.25 h. Absorption was gradual. For the highest dose, 100 mg, the mean C_max_ was 1289.8 ng/mL at a median T_max_ of 4.3 h. Mean AUC_0–24h_ was 11 275.1 h ng/mL. The mean half‐life ranged from 7.5 h with a 1‐mg dose, to 4.3 h with a 100‐mg dose with a tendency for a shorter apparent terminal half‐life with dose escalation up to 12 mg, which increased at higher doses. Variability in exposure between participants increased with dose escalation, with the greatest increases seen between 48 and 100 mg; 57.2% CV for C_max_ and 60.4% CV for AUC_0–24h_ compared to 16.7% CV for C_max_ and 23.3% CV for AUC_0–24h_ at 1‐mg dose.

### 3.3 MAD

**FIGURE 3:**
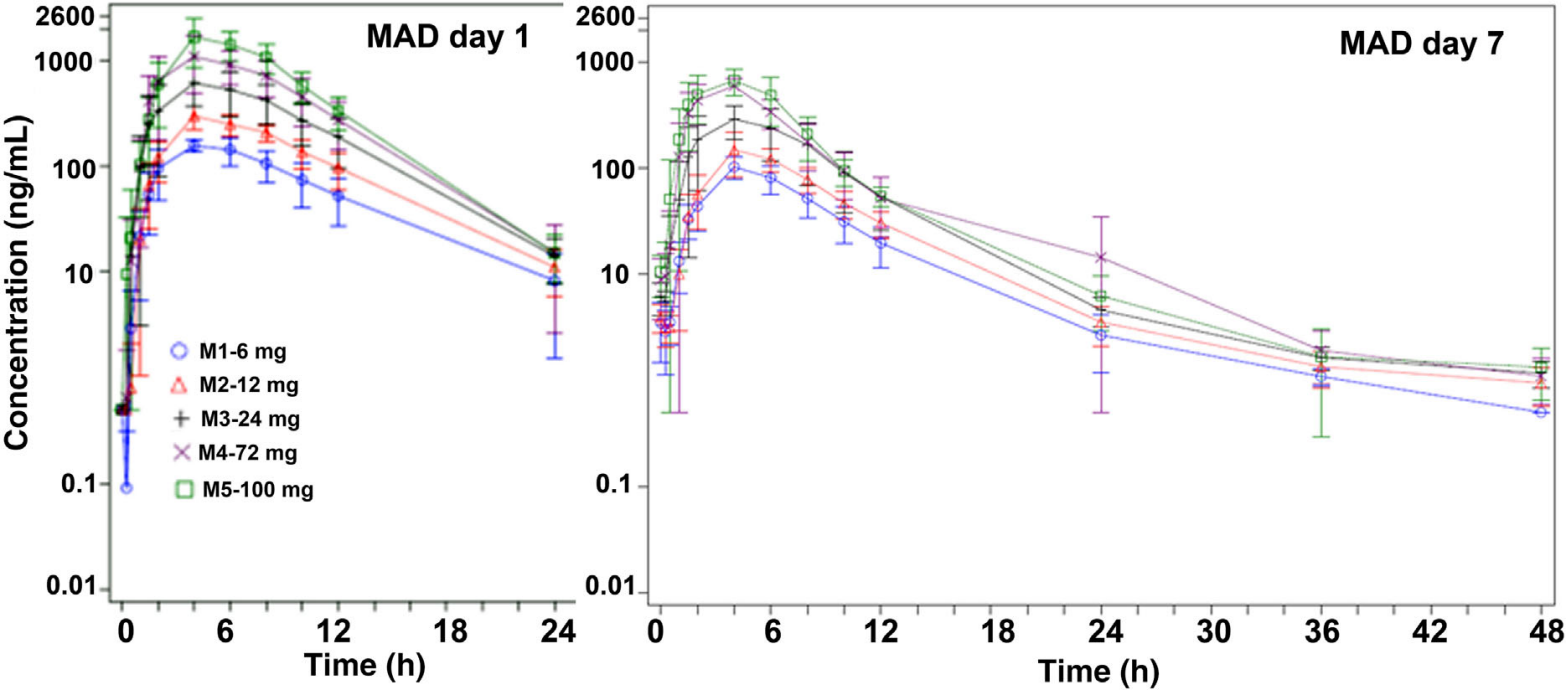
Overlaid mean plasma KCL‐286 concentration *vs.* nominal time (log‐linear scale) by treatment group (pharmacokinetic set)—multiple ascending dose (MAD). Error bars denote standard deviation.

Plasma concentration–time profiles overlaid for each dose group of the MAD cohorts are in Figure 3 (log‐linear scale Day 1 and Day 7). Plasma PK parameters following multiple ascending oral doses are summarized for Day 1 and Day 7 in Table 3.

**TABLE 3:** Summary of pharmacokinetic parameters (PK set)—multiple ascending dose.

Parameter	Unit	Treatment dose				
Plasma		M1, 6 mg (*n* = 6)	M2, 12 mg (*n* = 6)	M3, 24 mg (*n* = 6)	M4, 72 mg (*n* = 6)	M5, 100 mg (*n* = 6)
AUC_0‐inf_, D1	h ng/mL	1600.3 (436.2)	2801.4 (721.89)	5795.1 (1979.4)	9551.1 (4018.2)	12 998.1 (4375.7)
AUC_0‐inf_, D7	h ng/mL	789.9 (222.71)	1181.5 (229.11)	2391 (688.70)	3802.5 (776.64)	4390.8 (604.18)
AUC_0‐t_ D1	h ng/mL	1544.6 (383.87)	2738.0 (702.99)	5714.3 (2030.41)	9483.2 (4005.02)	12 931.7 (4426.6)
AUC_0‐t_ D7	h ng/mL	792.2 (221.60)	1169.8 (298.37)	2379.3 (689.69)	3791.8 (769.74)	4380.2 (601.87)
C_max_ D1	ng/mL	173 (28.64)	303.3 (74.48)	649.7 (265.99)	1169.51(530.63)	1760.3 (729.44)
C_max_ D7	ng/mL	103.8 (24.40)	158.3 (62.45)	342.7 (97.96)	602.8 (108.56)	766.7 (92.26)
T_1/2_ D1	h	4.150 (0.7535)	3.761 (0.5291)	3.516 (1.3654)	2.771 (0.4663)	2.752 (0.7606)
T_1/2_ D7	h	4.358 (0.866)	8.655 (4.2241)	7.404 (3.3064)	6.453 (3.2255)	5.278 (1.7829)
λz D1	1/h	0.1719 (0.03274)	0.1875 (0.0273)	0.2137 (0.05124)	0.2553 (0.03676)	0.2639 (0.05257)
λz D7	1/h	0.1647 (0.03438)	0.1016 (0.0546)	0.1076 (0.03773)	0.1273 (0.04945)	0.1409 (0.03474)
Tlag D1	h	0.42 (0.342)	0.38 (0.140)	0.29 (0.102)	0.29 (0.246)	0.25 (0.158)
T_max_ D1	h	4.667 (1.0328)	4.367 (0.8042)	4.333 (0.8165)	4.333 (0.8165)	4.678 (1.0437)
T_max_ D7	h	4.35 (0.8093)	4.669 (1.0307)	4.253 (2.0945)	4.333 (0.8165)	3.678 (1.5218)
AUC_0–24h_ D1	h ng/mL	1545.3 (384.41)	2738.9 (703.27)	5715.3 (2030.21)	9484.4 (4005.35)	12 932.8 (4426.2)
AUC_0–24h_ D7	h ng/mL	773.7 (209.18)	1130.7 (298.84)	2323.6 (683.69)	3676.9 (758.29)	4319.3 (597.38)
Rac AUC_0–24 h_		0.500 (0.0486)	0.412 (0.0454)	0.440 (0.1539)	0.412 (0.0722)	0.405 (0.2538)
Rac C_max_		0.598 (0.1109)	0.512 (0.1308)	0.638 (0.3859)	0.562 (0.1277)	0.558 (0.4014)
SR (AUC)		0.482 (0.0467)	0.403 (0.0441)	0.430 (0.1316)	0.408 (0.0688)	0.400 (0.2422)
Urine						
Ae D1	ng	57.2 (140.03)	76.1 (186.39)	168.3 (186.63)	1222.9 (1434.5)	2730.6 (1847.21)
Ae D7	ng	36.8 (90.11)	38.4 (94.15)	247.8 (292.94)	1509.7 (766.53)	2801.1 (1952.76)
Ae_0–24h_ D7	ng	55.18(135.167)	38.44 (94.154)	238.55(276.683)	1497.5(853.44)	2905 (2063.758)

Abbreviations: Ae, KCL‐286 excreted unchanged in a collection period; Ae_0‐t_, KCL‐286 excreted unchanged to the last measurable collection point; Ae%, percent of the dose excreted unchanged; AUC, area under the plasma concentration‐time curve; AUC (_0‐inf_), AUC from dosing time extrapolated to infinity; AUC (_0‐t_), AUC from dosing time to last observation; C_max_, maximum observed concentration, occurring at T_max_; CL/F, clearance over F (bioavailability); CV, coefficient of variation; D, days; h, hours; T_lag_, time prior to the first measurable (nonzero) concentration; T_max_, time of C_max_; Vz/F, volume of distribution associated with the terminal phase divided by F (bioavailability); λz, first order rate constant associated with the terminal (log‐linear) portion of the curve.

Data show mean and standard deviation in brackets.

Mean exposure for the highest dose 100 mg, on Day 1 (mean C_max_ = 1760.3 ng/mL and mean AUC_0–24h_ = 12 932.8 h ng/mL) was 48 and 52% (for C_max_ and AUC_0–24_, respectively) of the PK stopping criteria (C_max_ of 3700 ng/mL and AUC_0–24h_ of 25 100 h ng/mL). At Day 7, the C_max_ and AUC_0–24h_ were 43 and 33% of the values on Day 1 (mean C_max_ 766.7 ng/mL and mean AUC_0–24_ 4319.3 h ng/mL).

These reductions in exposure from Day 1 to Day 7 were observed at all doses. Variability in exposure between participants for the MAD cohorts on Day 1 was low‐moderate (CVs of 16.6–41.4% for C_max_ and CVs of 24.9–42.4% for AUC_0–24h_) and interparticipant variability on Day 7 was low (CVs 23.5–12% for C_max_ and CVs 27–13.8% for AUC_0–24h_).

Urine PK concentration–time profiles are presented in Table 3. With increasing dose, there was corresponding increase in excretion of KCL‐286, which reached a maximum of Ae_0–24h_ Day 7 2905 ± 2063.758 ng in the MAD 5 cohort.

### 3.4 Food effect

Plasma concentration–time profiles and plasma PK parameters following single oral doses of 6 mg KCL‐286 in fasted and fed participants are shown in Figure 2 and Table 4. There was a median lag time of 0.25–0.5 h from dosing to appearance of KCL‐286 in plasma. Lag time appeared to be longer when dosed with food with 3/6 participants showing a lag time >0.25 h in the fasted state (longest lag time 0.5 h) and 4/6 in the fed state (longest lag time 2.0 h). Absorption of KCL‐286 was slower when dosed after a high calorie/high fat meal. Median T_max_ in the fed state (6.288 ± 2.1351 h) was later than in the fasted state (4.343 ± 0.7417 h), representing a 50% increase in the presence of food. Peak concentration was on average lower when dosed with food; C_max_ was 192.9 ± 43 ng/mL fasted and 141.1 ± 23 ng/mL with food, representing on average a 27% reduction. Total exposure was only slightly reduced by dosing with food; the mean AUC_0–24_ was 1672.4 ± 404 h ng/mL fasted and 1402.9 ± 344 h ng/mL fed representing a 12% reduction. PK variability was similar whether KCL‐286 was dosed in the fed or fasted state (CV 22–24% fasted, 16–24% fed). The apparent elimination half‐life was unaffected by dosing with or without food.

**TABLE 4:** Summary of overall pharmacokinetic parameters (PK set)—food interaction.

Parameter	Unit	Treatment dose (6 mg)	
		Fasted (*n* = 7)	Fed (*n* = 7)
AUC _0‐inf_, D1	h ng/mL	1739.6 (453.18)	1510.0 (482.57)
AUC_0‐t_ D1	h ng/mL	1732.4 (449.51)	1499.6 (476.51)
Cl/F	L/h	3.615 (0.759)	4.227 (0.9486)
C_max_ D1	ng/mL	192.9 (43.18)	141.1 (23)
T_1/2_ D1	h	4.468 (1.1904)	4.327 (0.9539)
λz D1	1/h	0.1661 (0.04955)	0.1660 (0.03069)
Tlag D1	h	0.36 (0.134)	0.64 (0.675)
T_max_ D1	h	4.343 (0.7417)	6.288 (2.1351)
Vz/F obs	L	22.797 (6.2870)	25.547 (4.6149)
AUC_0–24h_ D1	h ng/mL	1672.4 (404.63)	1402.9 (344.62)
CL/F (norm),	L/h/kg	0.050 (0.0119)	0.057 (0.0160)
Vz/F (norm),	L/kg	0.308 (0.0700)	0.341 (0.0495)

Abbreviations: Ae, KCL‐286 excreted unchanged in a collection period; Ae_0‐t_, KCL‐286 excreted unchanged to the last measurable collection point; Ae%, percent of the dose excreted unchanged; AUC, area under the plasma concentration‐time curve; AUC (_0‐inf_), AUC from dosing time extrapolated to infinity; AUC (_0‐t_), AUC from dosing time to last observation; C_max_, maximum observed concentration, occurring at T_max_; CL/F, clearance over F (bioavailability); CV, coefficient of variation; D, days; h, hours; T_lag_, time prior to the first measurable (nonzero) concentration; T_max_, time of C_max_; Vz/F, volume of distribution associated with the terminal phase divided by F (bioavailability); λz, first order rate constant associated with the terminal (log‐linear) portion of the curve.

Data show mean and standard deviation in brackets.

### 3.5 Assessment of dose proportionality

#### 3.5.1 SAD (includes 6‐mg dose from the food interaction substudy)

Increase in mean plasma exposure was between proportional and subproportional with dose in terms of mean C_max_ and AUC_0–24h_ which continued the trend to subproportionality observed at 48 mg in relation to lower doses (Table 2 and Table S3). This is likely to be partly a consequence of using 4 mg as the reference dose for visualizing proportionality over the entire dose range tested so far. Exposure was essentially dose‐proportional (within 2‐fold of the mean) from 2 to 48 mg. In general, the increase in mean C_max_ and AUC_0–24h_ shows a trend to subproportionality with dose. Taken together, these data may indicate a progressive decrease in the fraction of the total dose absorbed at higher doses.

For the 6‐mg fasted dose from the FI substudy, dose‐proportionality exposure increase was within 2‐fold of the nominal dose increment, and was considered to be effectively dose proportional and within normal variability (Table 4 and Table S4).

#### 3.5.2 MAD

For the MAD cohorts, across the entire dose range steady‐state concentration was achieved from Day 3 or earlier at predose. Low concentrations of KCL‐286 were detected around 1 ng/mL from 36 or 48 h postdose through to the follow‐up time (nominally 168 h after dose 7). Over the entire dose range investigated there was a trend to subproportional exposure with increasing dose on Day 1 of the MAD being essentially similar to that observed from the SAD up to 100‐mg single dose. A similar relationship was apparent on Day 7 of the MAD, but at plasma exposures approximately half that found on Day 1 (Table 3 and Table S3).

#### 3.5.3 Urine: All parts of the study

The amount of drug secreted in urine was proportional to the dose administered throughout all participants of the study (Tables 2 and 3).

### 3.6 PD results

#### 3.6.1 Exploratory biomarker for target engagement

**FIGURE 4:**
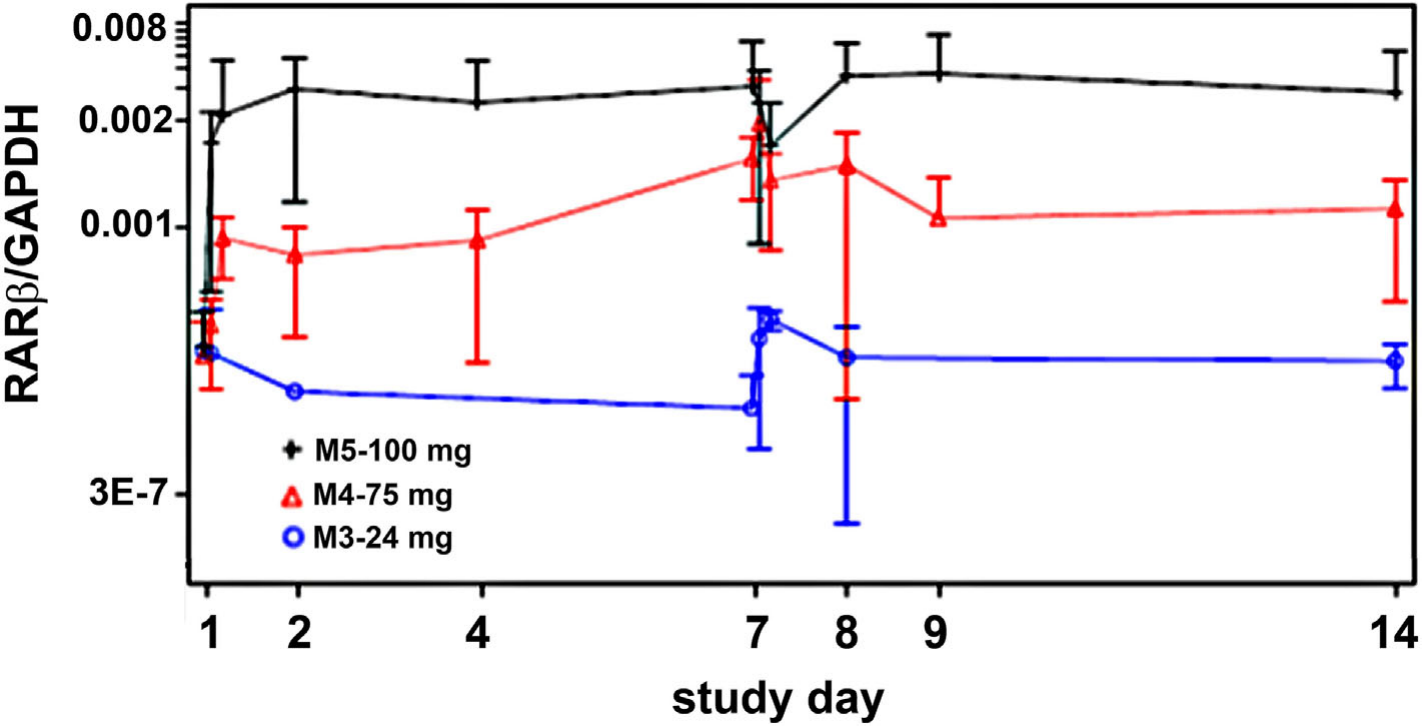
Expression of retinoic acid receptor (RAR)β2 in white blood cells in multiple ascending dose cohorts. With increasing dose of KCL‐286 there is a corresponding increase of RARβ2, which is maintained at least until Day 14, final day of blood collection. Error bars denote standard deviation.

RARβ2 was not detected in WBCs isolated from plasma from placebo samples or the 1‐ and 12‐mg dosed MAD cohorts. In the MAD cohorts of 24, 75 and 100 mg dosing, RARβ2 expression upon ligand binding becomes upregulated and measurable (Figure 4). There is a correlation between increase in RARβ expression and dose of KCL‐286 and plasma exposures, at least until Day 14, the last timepoint measured (Figure 4).

### 3.7 Safety evaluation

An overview of treatment‐emergent AEs (TEAEs) is provided for SAD in Table S5, for MAD in Table S6 and FI in Table S7. There were no TEAEs leading to death or serious AEs for any participants.

Across the SAD substudy, 56 TEAEs were reported by 31/56 (55.4%) participants. No dose relationship for any TEAE was evident. The most common TEAEs reported in the KCL‐286 treated participants included abnormal dreams (3 participants), skin exfoliation (3 participants), medical device site irritation (3 participants), headache (2 participants), dry eye (2 participants), dry skin (2 participants) and injection site bruising (2 participants). The most common TEAEs in placebo participants were abnormal dreams and headache (each in 2 participants). All other TEAEs were reported by no more than 1 participant. The events of medical device site irritation and injection site bruising resulted from study procedures (electrocardiogram electrodes and cannulation for blood draws). There was 1 clinically significant laboratory event of mild and reversible transaminase increase reported as a TEAE in a participant treated with a single 12‐mg dose.

One participant in the 100‐mg cohort reported a mild unrelated TEAE of visual field defect on Day 2 that resolved within an hour. In the FI substudy, 8 TEAEs were reported by 6/7 (85.7%) participants during the fasted period and 2 TEAEs were reported by 2/7 (28.6%) participants during the fed period. In the fasted period, TEAEs reported were headache (2 participants), dizziness, dysgeusia, epistaxis, nasal congestion, pain in extremity and skin exfoliation (each in 1 participant). In the fed period, TEAEs reported were dizziness and dry skin (each in 1 participant).

Across the MAD part of the study, 27 TEAEs were reported by 20/40 (50.0%) participants. The most common TEAEs reported in the KCL‐286 treated participants included dry skin (4 participants) and stomatitis (2 participants). The events of dry skin were reported in the 72‐ and 100‐mg multiple dosing groups and started within 6 days of dosing and resolved after 6–8 days. The events of stomatitis were reported in the 72‐mg multiple dosing group and started within 2 days of dosing and resolved after 11–12 days following nonpharmacological treatment. The most common TEAEs in placebo participants was dry skin (2 participants) that started on Day 2 and Day 10 and latest for 10–11 days. All other TEAEs were reported by no more than 1 participant. In the placebo group, there were 2 mild TEAEs reported for clinically significant laboratory events; these were mild hyperbilirubinaemia, which started on Day 3 and was still ongoing at follow‐up and was considered unrelated to IMP, and mild hypertriglyceridaemia, which started on Day 3 and resolved after 8 days and was considered possibly related to IMP.

TEAEs reported in participants excluded from the safety analyses were reported in 3/5 participants in the MAD substudy. Two TEAEs of dry skin were reported in 1 participant in the 6‐mg MAD group; 1 event started on Day 2 and the second event on Day 3; both were considered mild and possibly related to treatment and resolved after 9 and 12 days, respectively. One TEAE of ocular hyperaemia was reported in 1 participant in the 6 mg MAD group; the event started on Day 3 and lasted for 7 days. The TEAE of ocular hyperaemia was considered mild and possibly related to treatment. One TEAE of paraesthesia was reported in the same participant; the event started on Day 1 and lasted for 20 min. The TEAE of paraesthesia was considered mild and unrelated to treatment.

## 4 DISCUSSION

The treatment of nerve injuries including SCI is a major unmet clinical need as there are no therapies that can lead to axonal regeneration and functional recovery. RARβ2 activation is an attractive target to treat an SCI, as it is a neuronal located transcription factor that has multiple effects on pathways involved in axonal regeneration^12^, ^13^, ^20^, ^21^ as well as modulating the glial scar, which is a major inhibitory environment for functional recovery.^22^ KCL‐286 is a selective RARβ agonist that we have tested here to show its safety and tolerability in a first‐in‐man study.

The TEAEs observed are consistent with the known clinical safety of oral retinoids, such as bexarotene, acitretin, isotretinoin and alitretinoin, where it has been shown that they cause dry irritated and peeling skin, which once drug administration is stopped is fully reversible.^23^ The TEAEs associated with raised liver enzymes and eye disorders, which were observed in the SAD but not MAD study, are also known class effects of oral retinoids that are resolved after cessation of administration.^24^, ^25^, ^26^ Over the dose levels, frequencies and duration studied, no other clinically significant safety observations with KCL‐286 were seen. In fasted conditions compared with fed conditions, a single 6‐mg dose resulted in a higher frequency of nervous system disorders and 1 event of headache that led to study withdrawal. Headache is a known side effect of oral retinoids,^24^ however, headache was also reported in fasted placebo participants and at the dose levels and dosing frequencies studied, no TEAE of headache was considered related to the IMP.

The highest dose achieved in both the SAD and MAD cohorts was 100 mg, which surpasses the anticipated desired pharmacological exposures in the rat efficacy model.^15^ None of the clinical or PK stopping criteria were met during the study based on the preclinical dog 28‐day toxicology study. In general, there was an increase in mean C_max_ and AUC_0–24_ showing a trend to subproportionality with dose. The prolonged terminal elimination half‐life after repeat dosing has been described for other retinoids such as etretinate, and it could reflect intracellular storage (with subsequent extracellular release) of retinoids as metabolic lipid droplets, which accumulate in the nucleus in the nM range compared to μM range in the cytoplasm^27^ Consumption of a high calorie/high fat breakfast before receiving a dose of 6 mg KCL‐286 on average resulted in a slower absorption profile with a slightly reduced C_max_ but a similar overall exposure (AUC).

Concentrations of KCL‐286 were not measured in cerebrospinal fluid in this trial as concentrations in cerebrospinal fluid are a poor indicator of CNS penetrance^28^ and our preclinical package suggested that KCL‐286 can cross the blood brain barrier (BBB). The CNS penetrance has been determined for rat (1:1 BBB ratio), after oral administration of 10 mg/kg dose,^15^ which is much higher than the efficacy dose associated with the PAD in rat proof of concept studies. KCL‐286 may diffuse easily through the BBB due to its high lipophilicity but it may also be aided by a carrier transporter. Importantly, at the oral doses administered in the BBB study, which are higher than the efficacy dose, the CNS uptake is not saturated. In addition, p‐glycoprotein‐mediated transport does not seem to be a major mode of transport for KCL‐286, which, in the case of BBB permeation, is a well‐known factor to inhibit permeability due to its efflux activity. Therefore, it can be predicted that KCL‐286 will cross the BBB in humans at a not dissimilar rate to rats, although this will need to be confirmed in future trials.

Renal clearance of unchanged KCL‐286 is a minor elimination process. This may be due to a formation of other metabolites although in our preclinical studies of the potential for KCL‐286 (2 μM, 0.669 μg/mL) to be metabolized by a wide range of recombinant human cytochrome and UDP glucuronosyltransferases only CYP1A2 and UGT1A1 were found to metabolize KCL‐286 to an appreciable extent. Nevertheless, as KCL‐286 development advances in the clinic the identification of KCL 286 metabolites will be determined to confirm these findings.

Given that healthy volunteers were assessed in this study, CNS tissue could not be obtained to assess RARβ2 expression, so WBCs were used instead. The increase in RARβ2 expression does show that the drug engages its receptor. How much activation is required to elicit an effect in the injured nerve tissue is not known, but exposure has been achieved in the human healthy participants, which exceeds exposure in the injured rat to give a pharmacological response, suggesting that a clinical effect can be achieved in nerve injured patients. In addition, since a ceiling of RARβ2 expression was not achieved, it is possible that higher exposure may give a better therapeutic outcome.

The accumulation of the retinoid within the cell^27^, ^29^ with repeat dosing would be expected to maintain target engagement after drug cessation, and this is what is observed RARβ expression outlasts the treatment period by at least 7 days. It has also been shown that once a retinoid activates transcription it also initiates receptor catabolism, which allows a new RAR/RXR heterodimer to occupy the RARE using the same ligand.^30^ This extra period of biological activity may significantly enhance recovery after SCI. The KCL‐286 PK/PD results are highly encouraging and fully support its clinical therapeutic potential not only in SCIs but in a wider variety of clinical situations where regeneration of the CNS is desirable and of therapeutic significance; this includes stroke, traumatic brain injuries and multiple sclerosis.

## 5 CONCLUSIONS

This study has demonstrated that KCL‐286 an orally available RARβ agonist, engages its receptor, and the safety, tolerability, PK and PD data support the use of up to 100 mg daily in fed conditions for future clinical studies in SCIs and other CNS injuries where axonal regeneration is required.

## AUTHOR CONTRIBUTIONS

Jonathan P. T. Corcoran, Maria B. Goncalves, Adrian P. Mander, Tim Mant, Jane Holmes, Daryl Bendel, Jörg Täubel, John Posner, Henry Fok and Hana Hassanin made substantial contributions to the conception and design of the work and the analysis and interpretation of the data as well as drafting and revising the work. E.C. performed the reverse transcription–polymerase chain reaction. Tim Mant was the study sponsor chief investigator, Jörg Täubel was the principal investigator at Richmond pharmacology Ltd, and Daryl Bendel was the principal investigator at the University of Surrey. All authors have revised the work critically for important intellectual content and have provided final approval of the version to be published.

## CONFLICT OF INTEREST STATEMENT

The authors declare no competing financial interests. MBG and J.P.T.C have a matter of composition patent for KCL‐286.

## Supporting information


**TABLE S1** The predictive mean (95% CI) for $\mathrm{AU}C_{0-24}$ and C_max_ values for a new participant, for doses given to previous cohorts and also for potential next doses. It also shows the probability that any participant in the next cohort of 6 exceeds the maximum allowable exposure.
**TABLE S2** Summary of pharmacokinetic parameters at Day 1 (PK Set) – SAD
**TABLE S3** Evaluation of dose‐proportionality for PK parameters of KCL‐286
**TABLE S4** Evaluation of dose‐proportionality food effect for PK parameters of KCL‐286
**TABLE S5** Treatment‐emergent adverse event (safety set) SAD
**TABLE S6** Treatment‐emergent adverse event (safety set) MAD
**TABLE S7** Treatment‐emergent adverse event (safety set) FI

## Data Availability

The data in this article belong to the sponsor. The data may be made available to readers with permission from the corresponding author and sponsor.
