# Supplementary material for: Phase 1 safety, tolerability, pharmacokinetics and pharmacodynamic results of KCL‐286, a novel retinoic acid receptor‐β agonist for treatment of spinal cord injury, in male healthy participants
Source: Br J Clin Pharmacol. 2023 Aug 9;89(12):3573–83. doi: 10.1111/bcp.15854 (PMC10835503; doi:10.1111/bcp.15854)
Supplement: Supplementary file 1 — TABLE S1 The predictive mean (95% CI) for AUC0−24 and Cmax values for a new participant, for doses given to previous cohorts and also for potential next doses. It also shows the probability that any participant in the next cohort of 6 exceeds the maximum allowable exposure. TABLE S2 Summary of pharmacokinetic parameters at Day 1 (PK Set) – SAD TABLE S3 Evaluation of dose‐proportionality for PK parameters of KCL‐286 TABLE S4 Evaluation of dose‐proportionality food effect for PK parameters of KCL‐286 TABLE S5 Treatment‐emergent adverse event (safety set) SAD TABLE S6 Treatment‐emergent adverse event (safety set) MAD TABLE S7 Treatment‐emergent adverse event (safety set) FI [file BCP-89-3573-s001.docx]

**Phase 1 safety, tolerability, pharmacokinetics and pharmacodynamic results of KCL-286, a novel RARb agonist for treatment of spinal cord nerve injury, in male healthy participants**

**Maria B Goncalves^1^, Tim Mant^2^, Jörg Täubel^3*^, Earl Clarke^1^, Hana Hassanin^4^, Daryl Bendel^4^, Henry Fok^2^, John Posner^5^, Jane Holmes^6^, Adrian P Mander^7^, Jonathan P T Corcoran^1^**

1 Neuroscience drug discovery unit, The Wolfson Centre for Age-Related Diseases, King's College London, Guy's Campus, London SE1 1UL, UK

2 NIHR Biomedical Research Centre at Guy’s and St Thomas’ NHS Foundation Trust and King’s College London, Guy's and St Thomas' NHS Foundation Trust, 16th Floor, Tower Wing, Guy's Hospital, Great Maze Pond, London SE1 9RT, UK

3 Richmond Pharmacology Limited, 1A Newcomen Street, London Bridge, London SE1 1YR, UK

4 Surrey Clinical Research Centre, University of Surrey, Guildford, Surrey, GU2 7XH, UK

5 Centre for Pharmaceutical Medicine Research, Institute of Pharmaceutical Science, King's College London, 150 Stamford Street, London SE1 9NH, UK

6 Nuffield Department of Primary Care Health Sciences, University of Oxford, Oxford, UK

7 Centre for Trials Research, Cardiff University, 5th Floor Neuadd Meirionnydd, Heath Park, Cardiff. CF14 4YS, UK

**Correspondence**

Jonathan P. T. Corcoran, Neuroscience Drug Discovery Unit, The Wolfson Centre for Age‐Related Diseases, King's College London, Guy's Campus, London, UK.

Email: [jonathan.corcoran@kcl.ac.uk](mailto:jonathan.corcoran@kcl.ac.uk)

**Supplementary data**

**Methods**

**Analysis of RARβ2 expression in human WBCs**

Ten ml blood sample from each patient according to the standard operating procedure in tubes containing anticoagulant EDTA and RNAlater. Samples were centrifuged at ~1,500-2,000 ×g for 10-15 min at room temperature with no brake applied. Aliquots of plasma were then stored at -80°C until used. RNA was extracted (Qiagen 74104) and reverse transcription (Qiagen 205311) was carried out according to manufactures instructions. Primers used were human GAPDH and RARβ2. Samples were set up using a SYBR Green I Master mix (Roche 04707516001) and were carried out on a Roche lightcycler. Each reaction was run in triplicate and relative expression values obtained using a GAPDH standard curve. Primers used were, human gapdh forward gttcgtcatgggtgtgaacc and rev gcatggactgtggtcatgagt, product size 142 bp; human RARb2, forward tctacactgcgagtccgtct, and reverse tcaattgattgagcagtgtgc, product size, 105 bp. Products were identified by melting curve analysis

**An example using Bayesian statistics to predict dose for MAD cohort 5**

The fourth MAD cohort has been dosed and is based on 75 participants. The number of participants to be randomised to active dose in the next cohort is 6. Data is observed on day 1 for doses of 1, 2, 4, 6, 8, 12, 24, 48, 72 and 100mg, and on day 7 for doses of 6, 12, 24 and 72mg. Analysis is carried out using R version 4.0.3 (2020-10-10) and OpenBugs V3.2.3. Specifically, if P(At least one subject in the next cohort of patients has 𝐶_𝑚𝑎𝑥_ on either day> 3700|𝐷𝑎𝑡𝑎) < 𝛿_1_ and P(At least one subject in the next cohort of patients has 𝐴𝑈𝐶_(_0 − 24) on either day> 25100|𝐷𝑎𝑡𝑎) < 𝛿_1_, doses will be escalated to the next higher dose level. 𝛿_1_ = 0.05. The next higher dose-level is defined to be no more than 3 times the previous dose +5%. From this analysis (supplementary table 1), the highest dose that is suggested for the MAD cohort 5 that satisfies constraints for days 1 and 7 for both$AUC_{0-24}$ and $C_{max}$ is 105 mg.

**Supplementary Table 1**: The predictive mean (95% CI) for $AUC_{0-24}$ and C_max_ values for a new participant, for doses given to previous cohorts and also for potential next doses. It also shows the probability that any participant in the next cohort of 6 exceeds the maximum allowable exposure.

| **Dose** | **AUC_0-24_** | | | **C_max_** | | |
| --- | --- | --- | --- | --- | --- | --- |
|  | **Day 1** | **Day 7** | **Probability exceed threshold** | **Day 1** | **Day 7** | **Probability exceed threshold** |
| 1 | 382(201,718) | 254(129,498) | 0.000 | 41(20,81) | 31(15,66) | 0.000 |
| 2 | 635(338,1189) | 388(203,743) | 0.000 | 70(36,139) | 51(25,104) | 0.000 |
| 4 | 1056(567,1959) | 593(314,1118) | 0.000 | 121(62,235) | 82(41,163) | 0.000 |
| 6 | 1424(762,2644) | 760(405,1428) | 0.000 | 166(85,324) | 108(55,216) | 0.000 |
| 8 | 1759(943,3255) | 908(483,1699) | 0.000 | 208(106,403) | 133(67,262) | 0.000 |
| 12 | 2367(1269,4385) | 1164(627,2154) | 0.000 | 285(146,553) | 175(90,341) | 0.000 |
| 16 | 2924(1572,5426) | 1389(745,2576) | 0.000 | 357(183,694) | 214(109,417) | 0.000 |
| 24 | 3941(2118,7318) | 1779(951,3314) | 0.000 | 490(251,955) | 283(144,553) | 0.000 |
| 48 | 6548(3495,12198) | 2722(1454,5085) | 0.000 | 841(430,1643) | 457(231,901) | 0.000 |
| 72 | 8824(4727,16515) | 3491(1846,6621) | 0.004 | 1155(591,2271) | 605(302,1216) | 0.003 |
| 100 | 11225(5961,21036) | 4266(2242,8127) | 0.037 | 1492(761,2940) | 759(376,1531) | 0.029 |
| 105 | 11644(6192,21873) | 4392(2292,8400) | 0.048 | 1551(784,3066) | 784(385,1592) | 0.037 |
| 110 | 12054(6393,22697) | 4522(2371,8673) | 0.063 | 1610(814,3184) | 810(400,1643) | 0.049 |
| 115 | 12440(6621,23389) | 4649(2433,8893) | 0.082 | 1664(843,3288) | 836(413,1708) | 0.064 |
| 120 | 12842(6816,24101) | 4770(2485,9154) | 0.101 | 1722(873,3402) | 861(423,1758) | 0.078 |
| 140 | 14393(7631,26903) | 5243(2733,10138) | 0.212 | 1944(982,3847) | 957(468,1970) | 0.172 |

**Supplementary Table 2**: Summary of Pharmacokinetic Parameters at Day 1 (PK Set) – SAD

| Parameter | Unit | Treatment dose | | |  |
| --- | --- | --- | --- | --- | --- |
| Plasma |  | S1, 1 mg  N=3 | S5, 2 mg  N=4 | S3, 4 mg  N=6 | |
| AUC _0‑inf_, D1 | h.ng/mL | 389.2 (114.99) | 492.6 (181.02) | 1125.6 (259.07) | |
| AUC_0‑t_ D1 | h.ng/mL | 374.3 (115.24) | 465.3 (219.61) | 1117.6 (257.26) | |
| Cl/F | L/h | 2.713 (0.7379) | 4.728 (2.4709) | 3.694 (0.7414) | |
| C_max_ D1 | ng/ml | 35 (5.84) | 42.8 (13.03) | 110.9 (17.99) | |
| T_1/2_ D1 | h | 7.506 (2.2935) | 5.469 (0.8528) | 5.802 (0.7651) | |
| Lambda z D1 | 1/h | 0.0975 (0.02556) | 0.1293 (0.02173) | 0.1214 (0.0177) | |
| Tlag D1 | h | 0.58 (0.382) | 0.31 (0.239) | 0.34 (0.263) | |
| T_max_ D1 | h | 3.333 (1.1547) | 4.013 (0.025) | 4.342 (1.9749) | |
| Vz/F obs | L | 27.839 (1.823) | 35.196 (11.7668) | 30.652 (6.0795) | |
| AUC_0‑24h_ D1 | h.ng/mL | 334.1 (77.91) | 453.8 (158.31) | 1037.5 (225.66) | |
| CL/F (norm) | L/h kg | 0.035 (0.0119 | 0.062 (0.043) | 0.046 (0.0086) | |
| Vz/F (norm) | L/kg | 0.354 (0.0349) | 0.455 (0.2263) | 0.381 (0.0668) | |
|  |  |  |  |  | |

**Supplementary Table 3:** Evaluation of Dose-proportionality for PK Parameters of KCL-286

| **PK parameter (units)** | **Cohort** | **Exponent of the power model** | |
| --- | --- | --- | --- |
|  |  | **Estimate** | **90% CI** |
| AUC_(0-inf)_ (ng*h/mL) | SAD | 0.7506 | (0.6862, 0.8150) |
| C_max_ (ng/mL) | SAD | 0.8069 | (0.7431, 0.8707) |
| AUC_(0-inf)_ (ng*h/mL) | MAD day1 | 0.7097 | (0.6061,0.8133) |
| C_max_ (ng/mL) | MAD day 1 | 0.7708 | (0.6523,0.8893) |
| AUC_(0-inf)_ (ng*h/mL) | MAD day 7 | 0.6249 | (0.5485,0.7013) |
| C_max_ (ng/mL) | MAD day 7 | 0.7298 | (0.6465,0.8132) |

A power model was fitted with log (PK parameter) as response variable and log(dose) as predictor. AUC_0-inf_, area under the plasma concentration-time curve from zero hours to infinity; CI, confidence interval; C_max_, maximum plasma concentration; PK, pharmacokinetic.

**Supplementary Table 4:** Evaluation of Dose-proportionality Food Effect for PK Parameters of KCL-286

| PK parameter | **GM mean ratio**  **Fed/fasted** | **90% CI** |
| --- | --- | --- |
| AUC_(0-inf)_ (ng*h/mL) | 0.874 | (0.769,0.992) |
| AUC(0-t)[ng*h/mL] | 0.871 | (0.768,0.987) |
| C_max_ (ng/mL) | 0.716 | (0.601,0.854) |

A mixed effect model was fitted with log (PK parameter) as response variable, the food status as fixed effect and the subject as random effect. AUC_0-inf_, area under the plasma concentration-time curve from zero hours to infinity; AUC(0-t), area under the plasma concentration-time curve from zero hours to last measurable concentration; CI, confidence interval; C_max_, maximum plasma concentration; PK, pharmacokinetic. The exponent for AUC_(0-inf)_ is close to 1, which can be interpreted as a dose proportional relationship.

**Supplementary Table 5:** Treatment-Emergent Adverse Event (Safety Set) SAD

| Category | S1 , 1 mg  N=3  n (%) E | S2 - 2 mg  N = 4  n (%) E | S3 - 4 mg  N = 6  n (%)E | S4 - 8 mg  N = 6  n (%) E | S5 - 12 mg  N = 6  n (%) E | S6 - 24 mg  N = 6  n (%) E | S7 - 48 mg  N = 5  n (%) E | S8 - 100 mg  N = 6  n (%) E | Placebo  N =14  n (%) E | Total  N = 56  n (%) E |  |
| --- | --- | --- | --- | --- | --- | --- | --- | --- | --- | --- | --- |
| Any TEAE | 2 (66.7) 4 | 2(50.0) 2 | 3(50.0) 9 | 4 (66.7) 6 | 5 (83.3) 8 | 3 (50.0)7 | 4 (80.0) 6 | 2(33.3) 2 | 6(42.9) 12 | 31(55.4) 56 |  |
| Any Serious TEAE | 0 | 0 | 0 | 0 | 0 | 0 | 0 | 0 | 0 | 0 |  |
| Any TEAE Leading to Withdrawal | 0 | 0 | 0 | 0 | 0 | 0 | 0 | 0 | 0 | 0 |  |
| Any TEAE Leading to Death | 0 | 0 | 0 | 0 | 0 | 0 | 0 | 0 | 0 | 0 |  |
| TEAE by Relationship to IMP  Related | 0 | 1 (25.0) 1 | 1 (16.7) 2 | 1 (16.7) 1 | 1 (16.7) 1 | 1 (16.7) 1 | 3 (60.0) 3 | 0 | 3 (21.4) 3 | 11 (19.6) 12 |  |
| Not Related | 2 (66.7) 4 | 1 (25.0) 1 | 3 (50.0) 7 | 4 (66.7) 5 | 4 (66.7) 7 | 3 (50.0) 6 | 2 (40.0) 3 | 2 (33.3) 2 | 5 (35.7) 9 | 26 (46.4) 44 |  |
| TEAE by Severity  Mild | 2(66.7) 4 | 2 (50.0) 2 | 3 (50.0) 9 | 4 (66.7) 6 | 5 (83.3) 8 | 3 (50.0) 7 | 4 (80.0) 5 | 2 (33.3) 2 | 6 (42.9) 12 | 31(55.4) 55 |  |
| Moderate | 0 | 0 | 0 | 0 | 0 | 0 | 1 (20.0) 1 | 0 | 0 | 1 (1.8) 1 |  |
| Severe 0 0 0 0 0 0 0 0 0 0 | | | | | | | | | | | |

Abbreviation(s): AE - Adverse Event, IMP - Investigational medicinal product, TEAE - Treatment Emergent AE,

N - number of subjects at risk, n - number of subjects having an AE, E - number of events

Note(s): % - n/N*100

**Supplementary Table 6:** Treatment-Emergent Adverse Event (Safety Set) MAD

| Category | M1 - 6 mg  N =6  n(%)E | M2 - 12 mg  N=6 n(%)E | M3 - 24 mg  N =6 n(%)E | M4 - 72 mg  N =6 n(%)E | M5 - 100 mg  N =6 n(%)E | Placebo  N = 10  n (%) E | Total  N = 40  n (%) E |
| --- | --- | --- | --- | --- | --- | --- | --- |
| Any TEAE | 3 (50.0) 3 | 1 (16.7) 1 | 4 (66.7) 5 | 4 (66.7) 8 | 3 (50.0) 3 | 5 (50.0) 7 | 20 (50.0) 27 |
| Any Serious TEAE | 0 | 0 | 0 | 0 | 0 | 0 | 0 |
| Any TEAE Leading to Withdrawal | 0 | 0 | 0 | 0 | 0 | 0 | 0 |
| Any TEAE Leading to Death | 0 | 0 | 0 | 0 | 0 | 0 | 0 |
| TEAE by Relationship to IMP  Related | 0 | 0 | 0 | 1 (16.7) 1 | 1 (16.7) 1 | 0 | 2 (5.0) 2 |
| Not Related | 3 (50.0) 3 | 1 (16.7) 1 | 4 (66.7) 5 | 3 (50.0) 7 | 2 (33.3) 2 | 5 (50.0) 7 | 18 (45.0) 25 |
| TEAE by Severity  Mild | 3 (50.0) 3 | 1 (16.7) 1 | 4 (66.7) 5 | 4 (66.7) 8 | 3 (50.0) 3 | 5 (50.0) 7 | 20 (50.0) 27 |
| Moderate | 0 | 0 | 0 | 0 | 0 | 0 | 0 |
| Severe | 0 | 0 | 0 | 0 | 0 | 0 | 0 |

Abbreviation(s): AE - Adverse Event, IMP - Investigational medicinal product, TEAE - Treatment Emergent AE,

N - number of subjects at risk, n - number of subjects having an AE, E - number of events

Note(s): % - n/N*100

| Period 1 | | | Period 2 | | Overall | |
| --- | --- | --- | --- | --- | --- | --- |
| Category | 6 mg Fasted  N = 4  n (%) E | 6 mg Fed  N = 4  n (%) E | 6 mg Fasted  N = 3  n (%) E | 6 mg Fed  N = 3  n (%) E | 6 mg Fasted  N = 7  n (%) E | 6mgFed  N = 7  n (%)E |
| Any TEAE | 4 (100.0) 6 | 1 (25.0) 1 | 2 (66.7) 2 | 1 (33.3) 1 | 6 (85.7) 8 | 2 (28.6) 2 |
| Any Serious TEAE | 0 | 0 | 0 | 0 | 0 | 0 |
| Any TEAE Leading to Withdrawal | 1 (25.0) 1 | 0 | 0 | 0 | 1 (14.3) 1 | 0 |
| Any TEAE Leading to Death | 0 | 0 | 0 | 0 | 0 | 0 |
| TEAE by Relationship to IMP  Related | 0 | 0 | 0 | 0 | 0 | 0 |
| Not Related | 4 (100.0) 6 | 1 (25.0)1 | 2 (66.7) 2 | 1 (33.3) 1 | 6 (85.7) 8 | 2 (28.6) 2 |
| TEAE by Severity  Mild | 4 (100.0) 6 | 1 (25.0) 1 | 2 (66.7) 2 | 1 (33.3) 1 | 6 (85.7) 8 | 2 (28.6) 2 |
| Moderate | 0 | 0 | 0 | 0 | 0 | 0 |

| Severe | 0 | 0 | 0 | 0 | 0 | 0 |
| --- | --- | --- | --- | --- | --- | --- |

**Supplementary Table 7:** Treatment-Emergent Adverse Event (Safety Set) FI

Abbreviation(s): AE - Adverse Event, IMP - Investigational medicinal product, TEAE - Treatment Emergent AE,

N - number of subjects at risk, n - number of subjects having an AE, E - number of events

Note(s): % - n/N*100
